# Frailty and functional outcomes in patients with progressive fibrosing interstitial lung diseases receiving antifibrotic therapy: a real-life observational study

**DOI:** 10.3389/fmed.2026.1741725

**Published:** 2026-02-13

**Authors:** Domenica Francesca Mariniello, Fabio Perrotta, Carmine Picone, Vasiliki Liakouli, Stefano Sanduzzi Zamparelli, Giulia Maria Stella, Alfonso Reginelli, Gaetano Rea, Andrea Bianco, Vito D’Agnano

**Affiliations:** 1 Department of Translational Medical Sciences, University of Campania 'L. Vanvitelli', Naples, Italy; 2 U.O.C. Clinica Pneumologica L. Vanvitelli, Monaldi Hospital, A.O. dei Colli, Naples, Italy; 3 Istituto Nazionale Tumori di Napoli, IRCCS "G. Pascale", Naples, Italy; 4 Rheumatology Unit, Department of Precision Medicine, University of Campania Luigi Vanvitelli, Naples, Italy; 5 Division of Pneumology and Semi-Intensive Respiratory Therapy, A. Cardarelli Hospital, Naples, Italy; 6 Department of Internal Medicine and Medical Therapeutics, University of Pavia Medical School, Pavia, Italy; 7 Unit of Respiratory Diseases, Department of Cardiothoracic and Vascular, Istituto di Ricerca e Cura a Carattere Scientifico (IRCCS) Policlinico San Matteo, Pavia, Italy; 8 Department of Precision Medicine, University Hospital "Luigi Vanvitelli", University of Campania "Luigi Vanvitelli", Naples, Italy; 9 Department of Radiology, Azienda dei Colli, Monaldi Hospital, Naples, Italy

**Keywords:** antifibrotic therapy, frailty, idiopathic pulmonary fibrosis, nintedanib, pirfenidone, progressive fibrosing interstitial lung disease, progressive pulmonary fibrosis

## Abstract

**Objectives:**

Treatment of idiopathic pulmonary fibrosis (IPF) and progressive fibrosing interstitial lung disease (PF-ILD) remain challenging in elderly patients with frailty, which negatively impact outcomes. This study aims at describing the prevalence of frailty in a cohort of patients with PF-ILD on antifibrotic therapy and to investigate its potential impact on treatment effectiveness and tolerability.

**Methods:**

This monocentric, retrospective study enrolled a total of 64 patients with either IPF or other progressive pulmonary fibrosis (PPF) treated with antifibrotic treatment at our center between June 2022 and November 2023. The frailty status of patients with ILD was measured using the Clinical Frailty Scale (CFS). Baseline data were used to classify patients into two groups according to CFS: (1) non-frail patients with CFS < 5 or (2) frail patients with CFS ≥ 5.

**Results:**

The mean CFS score in the overall population was 5.02 ± 1.62. Thirty-seven (58%) were frail while 27 (42%) met criteria for no-frailty. Frail patients, compared to non-frail, were older (74.4 ± 4.66 vs. 70.6 ± 4.78, *p* = 0.004), and had significantly lower FVC (L) (2.31 ± 0.75 L vs. 2.78 ± 0.75 L, *p* = 0.03), percent predicted DLco (%DLco) (43.47 ± 13.52 vs. 54.6 ± 11.48, *p* = 0.003) and lower 6-min walk distance (6MWD) (305 ± 159 vs. 410 ± 94, *p* = 0.006) compared to no-frail patients at baseline. Frail patients had higher ILD-GAP index (4.62 ± 1.41 vs. 3.88 ± 1.18, *p* = 0.037) compared to non-frail patients. Interestingly, functional trajectories decline was not significantly different between frail and no-frail patients. Regarding safety profile, medication dose reduction due to adverse events was greater in frail patients (51.3% vs. 26%, *p* = 0.04) while not significant differences emerged in side effects.

**Conclusion:**

Frailty has been associated with poorer lung function and greater physical impairment in patients with fibrotic ILDs under antifibrotic treatment. Frail patients also more frequently require medication dose reduction due to adverse effects.

## 1 Introduction

Idiopathic pulmonary fibrosis (IPF) is the prototype of chronic and progressive fibrosing interstitial lung disease (PF-ILD) and it is strongly associated with advanced age, affecting more frequently subjects older than 60 years (1). The prevalence of other fibrotic ILDs similarly increases with age (2). Elderly patients with ILDs are more likely to have comorbidities, severe dyspnea and reduced quality of life, and experience medication side effects. At present, no curative pharmacological therapy is available for idiopathic pulmonary fibrosis (IPF). However, two antifibrotic agents, nintedanib and pirfenidone, have been approved for the treatment of IPF, as they have been shown to slow the rate of lung function decline (3–5). More recently, nintedanib has also been approved for the treatment of non-IPF progressive pulmonary fibrosis (PPF). Although these therapies are generally considered safe, they are frequently associated with adverse effects—particularly gastrointestinal symptoms such as nausea and diarrhea, as well as elevations in hepatic enzyme levels—which may lead to dose reduction or treatment discontinuation. In addition, nerandomilast has recently received FDA approval for the treatment of both IPF and PPF (6, 7).

Elderly patients are frequently taking multiple medications that may interact with the hepatic enzyme systems, such as CYP1A2, CYP3A4, P-glycoprotein, involved in the metabolism of antifibrotics. However, data on the tolerability and efficacy of antifibrotic therapies in elderly patients are limited as the elderly are commonly excluded from most clinical trials (5, 8).

Beyond chronological age, accelerated functional and biological aging are also critical determinants in patients with chronic respiratory diseases. Frailty is an age-associated state which leads to decreased strength, endurance, and physiological function resulting in an increased vulnerability to minor stressor events with negative outcomes (9, 10). As the world’s population ages, frailty is likely to increase in prevalence. Chronic lung diseases are known risk factors for the development of frailty, leading to increased health care utilization, hospitalizations, and mortality (11, 12). Prior or ongoing smoking exposure, hypoxemia, systemic inflammation, comorbidities, corticosteroid use, extrapulmonary symptoms such as fatigue and chronic pain, depression and physical inactivity due to dyspnea are common driver of accelerated aging and frailty in patients with chronic respiratory diseases (13–17). Furthermore, ILD patients frequently have biological features of accelerated aging, such as cellular senescence and accelerated telomere shortening (18, 19).

Biological and functional aging as well as frailty might cause faster pulmonary function decline in ILD patients, emerging as a better prognostic biomarker than a mere chronological age (20).

Several validated measures of frailty have been used for clinical and research purposes. Clinical Frailty Scale (CFS) is a simple tool to evaluate patients’ level of frailty, consisting of specific domain of comorbidity, function, and cognition, on a scale from 1(very fit) to 9 (terminally ill) (21–23).

Few studies have investigated the prevalence of frailty in ILD populations, and it remains unclear whether elderly and frail patients with fibrotic ILDs receive the same benefit from antifibrotics, and frailty may identify patients at high risk of complications from these drugs. Identification of frailty in patients with fibrotic ILDs might be useful for prognostication, more individualized management strategy, with careful consideration of medication adverse events.

We therefore aimed to describe the prevalence and the clinical implication of frailty in a cohort of patients with fibrotic ILD on antifibrotic therapy and to investigate its potential impact on treatment efficacy and tolerability.

## 2 Methods

### 2.1 Study design

This was a retrospective, single center, observational, real-life study conducted at “Interstitial Lung Diseases Centre - Clinica Pneumologica Vanvitelli, Ospedale Monaldi, Naples, Italy.” The study was approved by the local ethics committee board (AOC-0026569-2025) and was conducted in accordance with the Declaration of Helsinki and subsequent amendments. All patients provided written informed consent before participation.

### 2.2 Participants

Consecutive patients with a diagnosis of IPF or PPF admitted to our center between June 2022 and November 2023 were considered for study entry. The inclusion criteria were: (i) IPF or PPF diagnosed based on official ATS/ERS/JRS/ALAT clinical practice guideline criteria (53); (ii) age ≥ 65 years; (iii) Newly prescribed Nintedanib or Pirfenidone who had received antifibrotic therapy for at least 26 weeks from June 2022 to November 2023. All consecutive patients satisfying the inclusion criteria were retrospectively reviewed. Patients with active cancer or a hospitalization for acute exacerbation of ILD within the last 3 months were excluded.

Demographic and baseline characteristics were collected from the clinical record on enrolment. These included details on age, sex, body mass index (BMI), ILD diagnosis [according to the American Thoracic Society (ATS)/European Respiratory Society (ERS) classification (24)], comorbidities (COPD, asthma, heart failure, arterial hypertension, diabetes and dyslipidemia), smoking history, antifibrotic treatment (pirfenidone or nintedanib), concomitant immunomodulatory therapy, oxygen supplementation, lung function and exercise capacity. Pulmonary function tests (PFTs) were performed according to established guidelines, including measurement of spirometry as forced vital capacity (FVC) and forced expiratory volume in 1 s (FEV1), lung volumes and diffusion capacity of the lung for carbon monoxide (DLCO) (25–27). Exercise capacity was evaluated by 6-min walk test according to the official ATS/ERS technical standard for field walking tests in chronic respiratory diseases (28). ILD severity at baseline was characterized using the ILD gender-age-physiology (ILD-GAP) index, a multidimensional mortality risk prediction model based on the ILD diagnosis, sex, age, and physiological variables (FVC and DLCO) (29).

The frailty status of patients with ILD was measured using the Clinical Frailty Scale (CFS). CFS is a short and simple 9-point scale based on the clinical judgment of the professional using available clinical information with a time to complete <3 min. The domains assessed are nutritional status, physical activity, mobility, strength, energy, cognition, mood and social relations or social support. The scale is graded from 1 to 9 (1, very fit; 2, well; 3, managing well; 4, vulnerable; 5, mildly frail; 6, moderately frail; 7, severely frail; 8, very severely frail; 9, terminally ill). Baseline data were used to classify patients into two groups according to CFS: (1) non-frail patients with CFS < 5 or (2) frail patients with CFS ≥ 5 (22).

### 2.3 Endpoints

Endpoint of the study is two-fold: (i) to describe the prevalence of frailty in a cohort of patients with fibrotic ILD on antifibrotic therapy; (ii) to investigate the potential impact of frailty on treatment effectiveness and tolerability in patients with either CFS < 5 or with CFS ≥ 5. The pulmonary functions were obtained at baseline and 12 months after initiation of antifibrotic treatment. The rates of decline in FVC, DLCO, 6MWD after beginning of antifibrotic treatment were calculated by the difference between the FVC, DLCO, and 6MWD, 12 months after baseline and their values at baseline.

Adverse events (AEs) associated with antifibrotics were evaluated and the causes of discontinuation or dose reduction at 6 months after administration were assessed.

### 2.4 Statistical analysis

Demographic and baseline characteristics were collected from the clinical record on enrolment, anonymized and summarized in electronic record. All statistical analyses were performed using R Software (vers R 4.4.0). Demographic and clinical characteristics of the study population were summarized using mean, standard deviation, or counts and percentages. The Shapiro–Wilk test was used to assess the normality of data distribution; dependent and independent continuous variables were expressed as means with standard deviation and analyzed with Student’s *t*-test. The categorical variables were presented as a number or proportions and compared with the chi-square test or with Fisher’s exact test. Longitudinal changes in forced vital capacity (FVC%) were analyzed using linear mixed-effects models to account for within-subject repeated measurements. Frailty status, time (pre/post), sex, age, BMI and GAP index were included as fixed effects, with subject-specific random intercepts. Given the observational nature of the study, with the available cohort, the study had 80% power at a two-sided *α* level of 0.05 to detect a standardized between-group difference of Cohen’s *d* = 0.72 in unadjusted comparisons.

## 3 Results

A total of 64 patients with progressive fibrotic ILD newly treated with antifibrotics were enrolled. The mean (SD) age was 72.9 (± 5.12) years, 45 patients were males (70%).

The most common ILD diagnoses were IPF (*n* = 41, 64%) and systemic autoimmune disorders (SARDs-ILD) (*n* = 12, 19%). Characteristics of f-ILD participants in relation to their frailty status are provided in Table 1. PPF diagnoses were reported in Supplementary Table S1.

**Table 1:** Characteristics of participants according to frailty status.

Variable	Non-frails (CFS<5) n. 27	Frails (CFS≥5) n.37	*p*-value
Age, years	70.6 ± 4.78	74.4 ± 4.66	0.004
Gender Male (n, %)	20(44.4)	25(55.6)	0.392
BMI (kg/m^2^)	27.2 ± 4.34	27.13 ± 4.76	0.942
Smoking history (n, %)			0.927
Current	6 (22.2)	7 (18.9)	
Former	14 (51.9)	22(59.4)	
Never	7(25.9)	8(21.6)	
Fibrotic ILD			0.914
IPF	17(62.96)	24(64.86)	
PPF	10 (37.03)	13 (35.13)	
Antifibrotic			
Nintedanib	26(96.3)	30(81.1)	0.08
Pirfenidone	1(3.7)	7(18.9)	
T0 FVC (l)	2.78 ± 0.75	2.31 ± 0.75	0.03
T0 FVC % predicted	83.9 ± 15.2	75.2 ± 21.0	0.085
T0 DLCO% predicted	54.6 ± 11.48	43.47 ± 13.52	0.003
6MWD T0 (mt)	410 ± 94	305 ± 159	0.006
ILD-GAP INDEX	3.88 ± 1.18	4.62 ± 1.41	0.037
Respiratory failure (oxygen use)			0.06
LTOT at rest	0	5(13.5)	
SOT at exercise	9(33.3)	16(43.2)	
Comorbidities			
Pulmonary hypertension	1(3.7)	7(18.9)	
Arterial hypertension	15(55.5)	26(62.16)	
Heart Failure	1(3.7)	6(16.21)	
Diabetes Mellitus	7(25.9)	7(18.91)	
Dyslipidaemia	11(40.7)	15(40.5)	

Continuous variables are summarized by mean (standard deviation); categorical variables are summarized by count (percentage). BMI, body mass index; DLCO, diffusion capacity of the lung for carbon monoxide; FVC, forced vital capacity; GAP, gender, age and physiology; ILD, interstitial lung disease; IPF, idiopathic pulmonary fibrosis; LTOT, Long-term oxygen therapy; PPF, progressive pulmonary fibrosis; 6MWD, 6-min walk distance; SOT, Supplementary oxygen therapy.

Patients with SARDs-ILD received concomitant immunomodulatory therapy. Specifically, mycophenolate mofetil and abatacept were the most commonly co-prescribed immunosuppressive therapy (66%), while only two patients (16.7%) received azathioprine and 1 (8.33%) patient was treated with methotrexate (see Supplementary Table S2).

The mean CFS score in the overall population was 5.02 ± 1.62, with 37 (58%) patients met criteria for frailty and 27 (42%) met criteria for no-frailty.

The different frailty categories had similar sex, BMI, ILD diagnosis and smoking history but frail patients, compared to non-frail, were older (74.4 ± 4.66 vs. 70.6 ± 4.78, *p* = 0.004).

The most common comorbidities observed in our cohort were cardiovascular diseases, diabetes and dyslipidemia.

### 3.1 Pulmonary function of frail patients was worse compared to fit patients at baseline

At baseline, frail patients had significantly lower FVC (L) (2.31 ± 0.75 L vs. 2.78 ± 0.75 L, *p* = 0.03), and percent predicted DLco (%DLco) (43.47 ± 13.52 vs. 54.6 ± 11.48, *p* = 0.003) compared to no-frail patients. Also baseline %FVC was nominally despite not significant lower in frail patients compared to controls (75.2 ± 21 vs. 83.9 ± 15.2, *p* = 0.085).

Frail ILD patients, as compared to non-frail, had lower 6-min walking distance (6MWD) (305 ± 159 vs. 410 ± 94, *p* = 0.006). ILD-GAP index was significantly higher in the frail group versus the no frail group (4.62 ± 1.41 vs. 3.88 ± 1.18, *p* = 0.037).

Oxygen therapy at rest and at exercise was more frequently prescribed at baseline in the frail subgroup than the fit subgroup (56.7% vs. 33.3%, *p* = 0.06), although the difference was not significant.

### 3.2 Functional trajectories decline was not significantly different between frail and no-frail patients

**Figure 1:**
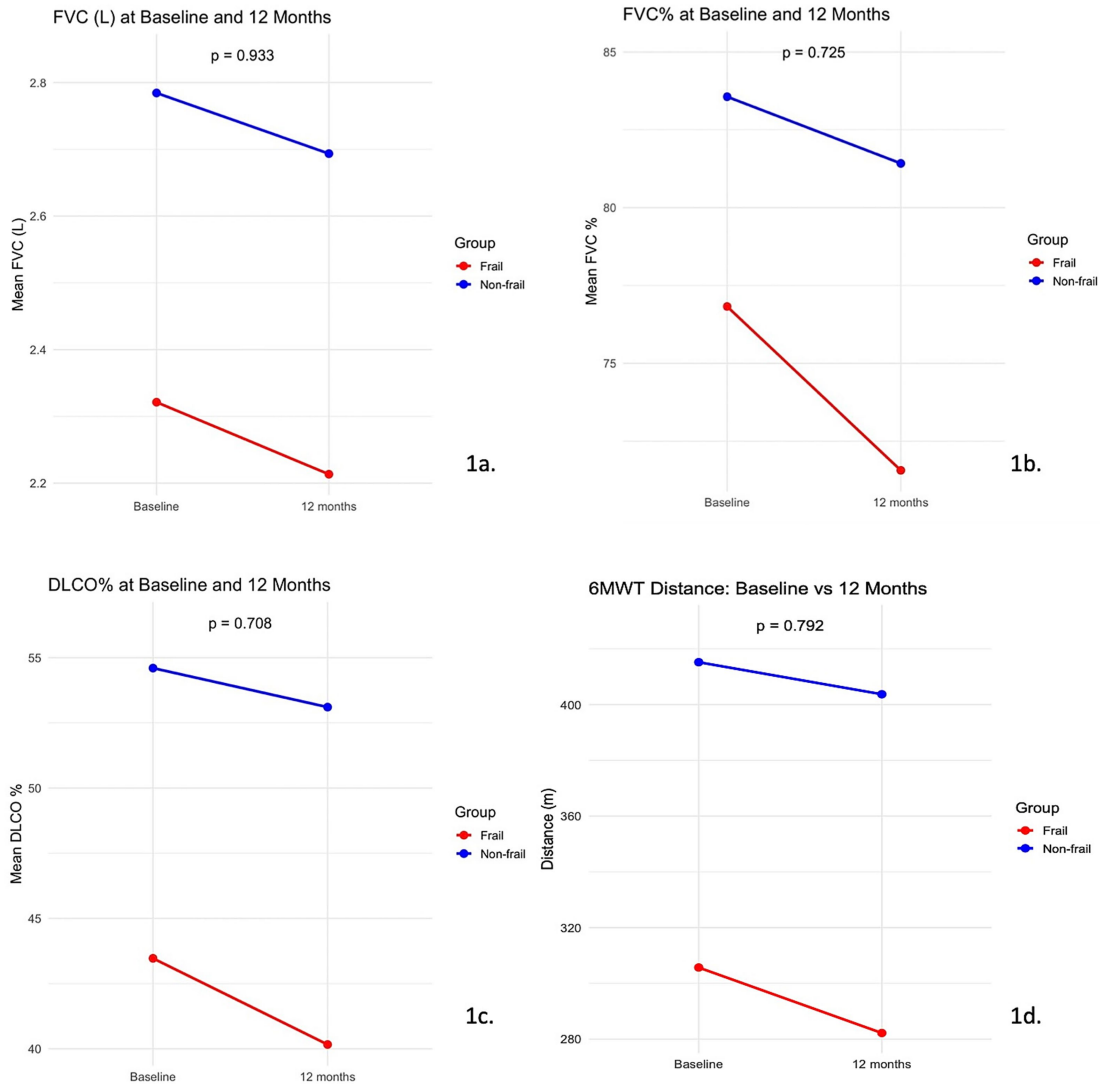
Functional trajectories decline in frail and no-frail patients. 6MWT, 6-min walk test; DLco, Diffusing capacity for carbon monoxide; FVC, forced vital capacity.

Patients in the frail subgroup similar declines in both FVC (L) [0.11 L (95% CI: −0.12 to 0.33) versus 0.09 L (95% CI: −0.26 to 0.44 L; *p* = 0.708), Figure 1a] and FVC % predicted (5.26; 95% CI, −6.79 to 20.16 vs. 2.14; 95% CI, −5.22 to 5.61, respectively; *p* = 0.725, Figure 1b) than patients in the fit subgroup.

Analogously, frail and non-frail patients had similar annual decline of DLCO % predicted [1.51% (95% CI: −4.5 to 7.5) versus 3.31% (95% CI: −4.62 to 11.23), *p* = 0.708, Figure 1c] and 6MWD [11.5 m (95% CI: −54.89 to 77.89 m) versus 23.5 m (95% CI: −43.09 to 90.09), *p* = 0.933, Figure 1d].

### 3.3 Medication dose reduction due to adverse events was greater in frail patients

The adverse event profile of antifibrotics was generally similar between frail subgroups. Table 2 shows the incidence of each adverse drug reaction to antifibrotic treatment that occurred within 12 months after administration in the 2 groups. Diarrhea was the most common event in both groups. Additionally, all adverse drug reactions were mild (Grade ≤ 2).

**Table 2:** Adverse events due to antifibrotic therapy in frail and no-frail patients.

Adverse events	Frail (*n* = 37)	No-frail (*n* = 27)	*p*-value
Any adverse event(s), n (%)	24 (65)	13 (48)	ns
Most frequent adverse events, n (%)			
Diarrhea	13 (35)	10 (37)	ns
Nausea	4 (11)	0	ns
Hepatic enzyme elevation	5 (13.5)	3 (11)	ns
Any adverse event(s) leading to			
Dose reduction, n (%)	19 (51.3)	7 (26)	*p* = 0.04
Discontinuation, n (%)	1 (2.7)	1 (3.7)	ns

GAP index, but not frailty status, is independently associated with longitudinal FVC%.

The proportion of patients who reduced dose due to adverse events was greater in frail patients than in the other group (51.3% vs. 26%, *p* = 0.04). However, the discontinuation rate due to all adverse drug reactions between the 2 groups was not significantly different (2.7% versus 3.7%, *p* = 0.99).

Longitudinal changes in FVC% have been analyzed using linear mixed-effects models to account for within-subject repeated measurements. The model (Table 3), refitted after removal of the non-significant interaction term (see Supplementary Table S3), showed that frailty status was not independently associated with longitudinal FVC%. In contrast, the GAP index was independently associated with FVC%, with higher scores corresponding to lower FVC% values.

**Table 3:** Fixed effects from the linear mixed-effects model for longitudinal FVC%.

Names	Effect	Estimate	SE	Lower	Upper	df	*t*	*p*
(Intercept)	(Intercept)	72.7237	3.604	65.661	79.787	43.7	20.1808	< 0.001
Frail	Yes—No	−0.1060	4.961	−9.829	9.617	41.8	−0.0214	0.983
Time	Pre—Post	1.0871	2.702	−4.209	6.383	15.7	0.4023	0.693
Sex	M—F	10.3758	7.965	−5.236	25.988	42.6	1.3026	0.200
Age	Age	−0.5567	0.463	−1.464	0.351	42.0	−1.2022	0.236
BMI	BMI	0.0396	0.511	−0.962	1.041	42.2	0.0775	0.939
GAP index	GAP index	−8.6299	2.426	−13.385	−3.875	42.1	−3.5574	< 0.001

The linear mixed-effects model included subject-specific random intercepts to account for within-subject repeated measurements and was adjusted for frailty status, time (pre/post), sex, age, BMI, and GAP index.

## 4 Discussion

Frailty is a medical syndrome characterized by decreased physiological reserve and resistance to stressors as consequence of accumulation of multiple deficits, leading to increased vulnerability and negative health outcomes (30); the prevalence of frailty gradually increases with age and in selected populations with specific chronic conditions (31).

In our study of adults aged over 65 years with progressive fibrotic ILDs, frailty is highly prevalent, suggesting that frailty’s assessment should be always considered in patient evaluation. In line with other studies, more than half (58%) of elderly patients with fibrotic ILDs in our cohort are frail (32, 33). In three studies comparing frailty prevalence in SARDs-ILD versus other ILDs, a higher frailty prevalence in SARDs-ILD was reported (34–36) and women are consistently reported to be at higher risk for frailty compared to men (31). Conversely, we did not find differences in ILD diagnosis or sex between the two subgroups.

A recent systematic review (37) assessed the prevalence and the impact of frailty in people with ILD using two different frailty scales: the Fried Frailty Phenotype (31) and the Frailty Index (22); the six studies included in the analysis showed a median prevalence of frailty in ILD patients of 48% and a trend toward worsening mortality for frailer people. In our study we used the Clinical Frailty Scale (CFS), a short and easy tool with a similar validity, which has been lately emerged as a valuable contributor to risk stratification in patients with fibrotic ILDs (22, 38, 39). In a cohort of 1,587 patients with fibrotic ILD, Guler and colleagues, using the Clinical Frailty Scale, found that frailty was a risk factor for early mortality (hazard ratio, 5.58; 95% CI, 3.64–5.76, *p* < 0.001) in the entire cohort (39). In addition, patients in the frail subgroup had larger annual declines in FVC % predicted than patients in the fit subgroup (2.32; 95% CI, 3.39–1.17 vs. 1.55; 95% CI, 2.04–1.15, respectively; *p* = 0.02) (39). We observed that patients in the frail group had larger declines in FVC %, consistent with the aforementioned study. However, this difference did not reach statically significance. This discrepancy may be explained by difference in sample size, as Guler and colleagues analyzed a substantially larger cohort.

In our cohort, frail patients have significant worse performance on exercise tolerance test with shorter 6MWD at baseline compared to fit patients (305 ± 159 vs. 410 ± 94; *p* = 0.006). In addition, frailty was commonly associated with objective markers of disease severity, such as worse ILD-GAP index and supplemental oxygen. In this respect, pulmonary rehabilitation programs and physical interventions may serve as additional support for these patients to improve physical activity, muscle strength and symptoms (40, 41).

Treatment discontinuation due to adverse effects (AEs) represents a clinically relevant incident for patients under antifibrotic treatment due to fibrotic ILDs, ranging from 23.8 to 46% in different cohort studies (11, 42). Regarding frail patients, our study demonstrated that antifibrotic therapies were slightly less tolerated compared to fit patients, leading to more dose reductions compared to fit patients [19 (51.3%) vs. 7 (26%), *p* = 0.04]. The relatively low discontinuation rates observed in our cohort are primarily explained by the study design, as we intentionally included only patients who had received antifibrotic therapy for at least 26 weeks. This inclusion criterion was adopted to ensure an adequate exposure period for evaluating treatment effectiveness, particularly in relation to longitudinal functional outcomes. We acknowledge that this selection may have excluded patients who discontinued treatment early due to intolerance, adverse events, or rapid disease progression, potentially leading to an underestimation of real-world discontinuation rates. Previous studies have evaluated the effectiveness and safety of antifibrotic drugs in elderly IPF patients (43–45). Data stemming from literature support that antifibrotics are effective also in elderly patients with IPF, although data on tolerability in this subset of patients are partly conflicting. By pooling data from five clinical trials on nintedanib, Glaspole and coworkers reported a higher rate of discontinuation rate due to adverse events in IPF patients aged ≥ 75 years than < 75 years (26.4% versus 16.0%) (46). Conversely, in a cohort of 65 patients with IPF, Komatsu et al. did not report a different rate of nintedanib discontinuation between IPF patients aged ≥75 versus those aged <75 years (47). Likewise, more recently, in another observational, retrospective, multicenter real-life study recruiting 159 IPF patients treated with nintedanib, FVC decline after 12 month, mortality, dose reduction or drug discontinuation were not significantly different between patients aged ≤ 80 versus >80 years (48) suggesting that frailty rather than older age may be a risk factor for dose reduction in patients with IPF.

From a clinical perspective, interaction between respiratory impairment ad functional frailty has crucial implication. Indeed, accumulating evidence indicates that respiratory impairment and pulmonary diseases are not only strongly associated with frailty, but the mortality risk is relevantly increased when these conditions are concomitant in patients with either chronic obstructive pulmonary disease (COPD) (49–52). In addition, adding frailty indices to traditional risk factors such as age, sex and smoking status, seems to improve the accuracy of mortality prediction in subjects with COPD (53).

We acknowledged that the study has limitations. The retrospective and single-center design represents a first limitation. Secondly, interpretation between the subgroups of ILD patients according to their frailty status should be made with caution due to the limited number of patients in each group. Moreover, the study may have not been sufficiently powered to detect small but potentially clinically relevant differences between groups. However, we recognized that our data are in line with other larger cohort studies. Prospective studies with larger numbers of patients are needed to evaluate the prognostic impact of frailty and to investigate the efficacy as well as the safety of antifibrotic treatment in these patients. Finally, our results should be interpreted as reflecting treatment effectiveness in patients who are able to sustain antifibrotic therapy beyond the early treatment period, rather than overall treatment persistence in an unselected IPF/PPF population.

## 5 Conclusion

Frailty is highly prevalent among elderly patient with fibrotic ILDs, and it has been associated with poorer lung function and greater physical impairment. Frail patients also more frequently require medication dose reduction due to adverse effects. In this respect, multidisciplinary management appears therefore essential, particularly in older patients. However, the lack of consensus on frailty assessment in these patients and the complexity of some frailty assessment tools have produced a substantial heterogeneity among studies, limiting their use in clinical practice. The real challenge is now to develop a standardized and pragmatic frailty definition and screening tool to allow a homogeneous assessment, risk stratification and treatment planning in the ILD population.

## Data Availability

The raw data supporting the conclusions of this article will be made available by the authors, without undue reservation.
