# Supplementary material for: Frailty and functional outcomes in patients with progressive fibrosing interstitial lung diseases receiving antifibrotic therapy: a real-life observational study
Source: Front Med (Lausanne). 2026 Feb 13;13:1741725. doi: 10.3389/fmed.2026.1741725 (PMC12946019; doi:10.3389/fmed.2026.1741725)
Supplement: Supplementary file 1 [file Table_1.docx]

| PPF diagnosis |  | Non-Frails (10) | Frails (13) |  |
| --- | --- | --- | --- | --- |
| RA-ILD |  | 3 (30%) | 3(23,07%) |  |
| *DIP* |  | 0 | 1 (7,69%) |  |
| F-HP |  | 1(10 %) | 2(15,38%) |  |
| *i*NSIP |  | 4(40 %) | 3(23,07%) |  |
| SSc-ILD |  | 0 | 2(15,38%) |  |
| SS-ILD |  | 2 (20%) | 2 (15,38%) |  |

**Table S1. PPF diagnoses.** DIP: Desquamative Interstitial Pneumonia; f-HP: Fibrotic hypersensitivity pneumonitis (HP); iNSIP: Idiopathic Nonspecific Interstitial Pneumonia; RA-ILD: Rheumatoid arthritis-interstitial lung disease; SS: Sjögren's syndrome; SSc-ILD: systemic sclerosis associated interstitial lung disease;
