# Supplementary material for: Frailty and functional outcomes in patients with progressive fibrosing interstitial lung diseases receiving antifibrotic therapy: a real-life observational study
Source: Front Med (Lausanne). 2026 Feb 13;13:1741725. doi: 10.3389/fmed.2026.1741725 (PMC12946019; doi:10.3389/fmed.2026.1741725)
Supplement: Supplementary file 2 [file Table_2.docx]

| Immunomudolatory therapy |  | Non-frails  n = 27 | Frails  n = 37 |
| --- | --- | --- | --- |
| *OCS* |  | 4 (14,81%) | 4 (10,81%) |
| *MMF* |  | 2 (7,40%) | 4(10,81%) |
| *Colchicine* |  | 0 | 1 (2,70%) |
| *Azathioprine* |  | 1 (3,70%) | 2 (5,40%) |
| *Abatacept* |  | 1 (3,70%) | 2 (5,40%) |
| *MTX* |  | 0 | 1 (2,70%) |

**Table S2. Immunomodulatory therapy in frails and non-frails.** MMF: Mycophenolate mofetil; OCS: Oral corticosteroids; MTX: Methotrexate.
