# Supplementary material for: Frailty and functional outcomes in patients with progressive fibrosing interstitial lung diseases receiving antifibrotic therapy: a real-life observational study
Source: Front Med (Lausanne). 2026 Feb 13;13:1741725. doi: 10.3389/fmed.2026.1741725 (PMC12946019; doi:10.3389/fmed.2026.1741725)
Supplement: Supplementary file 3 [file Table_3.docx]

| Names | Effect | Estimate | SE | Lower | Upper | df | t | p |
| --- | --- | --- | --- | --- | --- | --- | --- | --- |
| (Intercept) | (Intercept) | 71.7075 | 3.931 | 64.00 | 79.412 | 49.7 | 18.2418 | < .001 |
| Frail | Yes - No | -2.0130 | 6.815 | -15.37 | 11.345 | 49.4 | -0.2954 | 0.769 |
| Time | Pre - Post | 3.3670 | 4.154 | -4.78 | 11.509 | 12.1 | 0.8105 | 0.433 |
| Sex | M - F | 13.3285 | 8.971 | -4.25 | 30.911 | 51.2 | 1.4858 | 0.143 |
| Age | Age | -0.5475 | 0.475 | -1.48 | 0.384 | 40.3 | -1.1515 | 0.256 |
| BMI | BMI | 0.0416 | 0.560 | -1.06 | 1.140 | 40.1 | 0.0742 | 0.941 |
| GAP INDEX | GAP INDEX | -8.7045 | 2.498 | -13.60 | -3.809 | 40.5 | -3.4851 | 0.001 |
| Frail x Time | Yes-No x Pre-Post | 4.2407 | 5.968 | -7.46 | 15.938 | 12.9 | 0.7106 | 0.490 |
| Frail x Sex | Yes-No x M-F | 0.9868 | 12.972 | -24.44 | 26.411 | 42.6 | 0.0761 | 0.940 |
| Time x Sex | Pre-Post x M-F | -6.4557 | 8.711 | -23.53 | 10.618 | 11.9 | -0.7411 | 0.473 |

Table S3: Fixed effects from the linear mixed-effects model.
